# Satiety responsiveness following dietary exposure to ultra-processed foods differing in texture-derived eating rate: a secondary analysis of a randomized controlled crossover diet intervention

**DOI:** 10.1007/s00394-026-04111-7

**Published:** 2026-09-24

**Authors:** Marieke van Bruinessen, Zhen Liu, Lise A. J. Heuven, Markus Stieger, Marlou P. Lasschuijt, Ciarán G. Forde

**Affiliations:** Sensory Science and Eating Behavior Group, Division of Human Nutrition and Health, Wageningen University and Research, Wageningen, The Netherlands

**Keywords:** Satiety, Ultra-processed foods, Ghrelin, Pancreatic polypeptide, Peptide YY, Glucagon-like peptide-1, Eating rate

## Abstract

**Purpose:**

Disrupted satiety signalling has been proposed as a possible mechanism driving high energy intakes associated with the consumption of ultra-processed foods (UPFs). We examined whether transitioning from a diet low in UPFs to a high-UPF diet affects satiety responsiveness.

**Methods:**

In a randomized crossover trial, participants (n = 32; mean ± SD: age 27 ± 2 y; BMI 23.6 ± 2.3 kg/m^2^) with habitual low-UPF intakes completed two 14-day UPF diets (~ 95%EN from UPFs), separated by a 14-day washout period. Acute satiety responsiveness was assessed using a mixed-meal tolerance test with a fixed-calorie load at baseline and following each 14-day diet. Subjective appetite ratings and plasma concentrations of glucagon-like peptide-1 (GLP-1), ghrelin, pancreatic polypeptide (PP), and peptide YY (PYY) were measured from 10 min prior to 180 min following the fixed-calorie load. Satiety responsiveness was also assessed over-time within each UPF diet-arm, through daily fasting-state appetite ratings prior to breakfast.

**Results:**

A small but significant difference was observed in acute subjective satiety ratings for total area under the curves (AUCs), with higher hunger and lower fullness AUCs following the high-UPF diets compared with baseline-measurements, though no significant differences were observed at individual timepoints (all, *p* > 0.05). Satiety hormone responses to the fixed-calorie load did not differ between baseline and following 14-day high-UPF exposure (all, *p* > 0.05). Within each diet period, fasting-state appetite ratings remained stable across days on both UPF diets.

**Conclusion:**

Our results do not support the hypothesis that satiety responsiveness is disrupted following two 14-day periods of high-UPF diet exposure. The study was pre-registered at clinicaltrials.gov (NCT06113146).

**Supplementary Information:**

The online version contains supplementary material available at https://doi.org/10.1007/s00394-026-04111-7.

## Introduction

Impaired satiety signalling has been proposed as a potential mechanism that promotes excess energy intakes from diets high in ultra-processed foods (UPFs) [1, 2]. Ultra-processed foods are defined as ‘industrial formulations that are made entirely from food derivatives, chemical substances and a sequence of processes that bear little resemblance to the original food material’ [3]. Foods that fall within this category are thought to stimulate dietary energy intake through the promotion of hunger and their supposedly low satiety capacity [4]. Putative underlying mechanisms include destruction of the food matrix, presence of food additives and the higher reward value of UPFs [5–8]. Industrial changes to the structure of the food matrix are hypothesized to accelerate nutrient absorption and consequently blunt the release of appetite-suppressing hormones [6, 7, 9]. Food additives have been suggested to disrupt neuro-endocrine signals on the gut-brain axis and weakening the homeostatic satiety system [10–12]. This system is also thought to be disrupted or overruled by the high reward properties often attributed to UPFs, leading to sustained hunger in comparison to an equivalent calorie load from less processed foods [13, 14].

Dietary energy intake and satiety are influenced by multiple food characteristics, including nutrient composition, energy density, and textural properties. Accounting for these characteristics is therefore essential when drawing causal conclusions about the effects of industrial food processing on energy intake and satiety. [15–19]. Food textural properties and associated eating rates (ERs, g/min) are specifically important when assessing the satiety capacity of a food or food group [20]. Slower eating rates enhance satiety signalling through increased glucagon-like peptide-1 (GLP-1) and peptide YY (PYY) responses and greater suppression of ghrelin concentrations following a meal [21–25]. This leads to an earlier onset of satiation (i.e. reduced meal intake) and increased satiety (i.e. higher post-meal fullness per calorie consumed), thereby reducing food and energy intake during and after slowly consumed meals [26–28]. Therefore in this secondary-analysis [29, 30], we study the effect of exposure to diets high in UPFs with different textural properties on post-meal feelings of satiety and related satiety hormone signalling.

Our primary objective was to examine whether transitioning from a diet low in UPFs to a diet high in UPFs affects satiety responsiveness. The secondary objective was to determine whether eating rate of the diet (i.e. foods that can be consumed faster vs. foods that can be consumed slower) moderates satiety responsiveness to a UPF diet. We hypothesize that satiety responsiveness (subjective or hormonal) would remain unchanged from baseline following the transition to a diet high in UPFs, as measured both acutely (MMTT) and over time within each diet arm. For our secondary outcome, we hypothesized that satiety responses would be enhanced on a ‘Slow’ UPF diet compared to the ‘Fast’ UPF diet over the 14 days on each diet (i.e. higher fasting-state fullness ratings, lower hunger ratings).

## Methods

### Study design

**Fig. 1:**
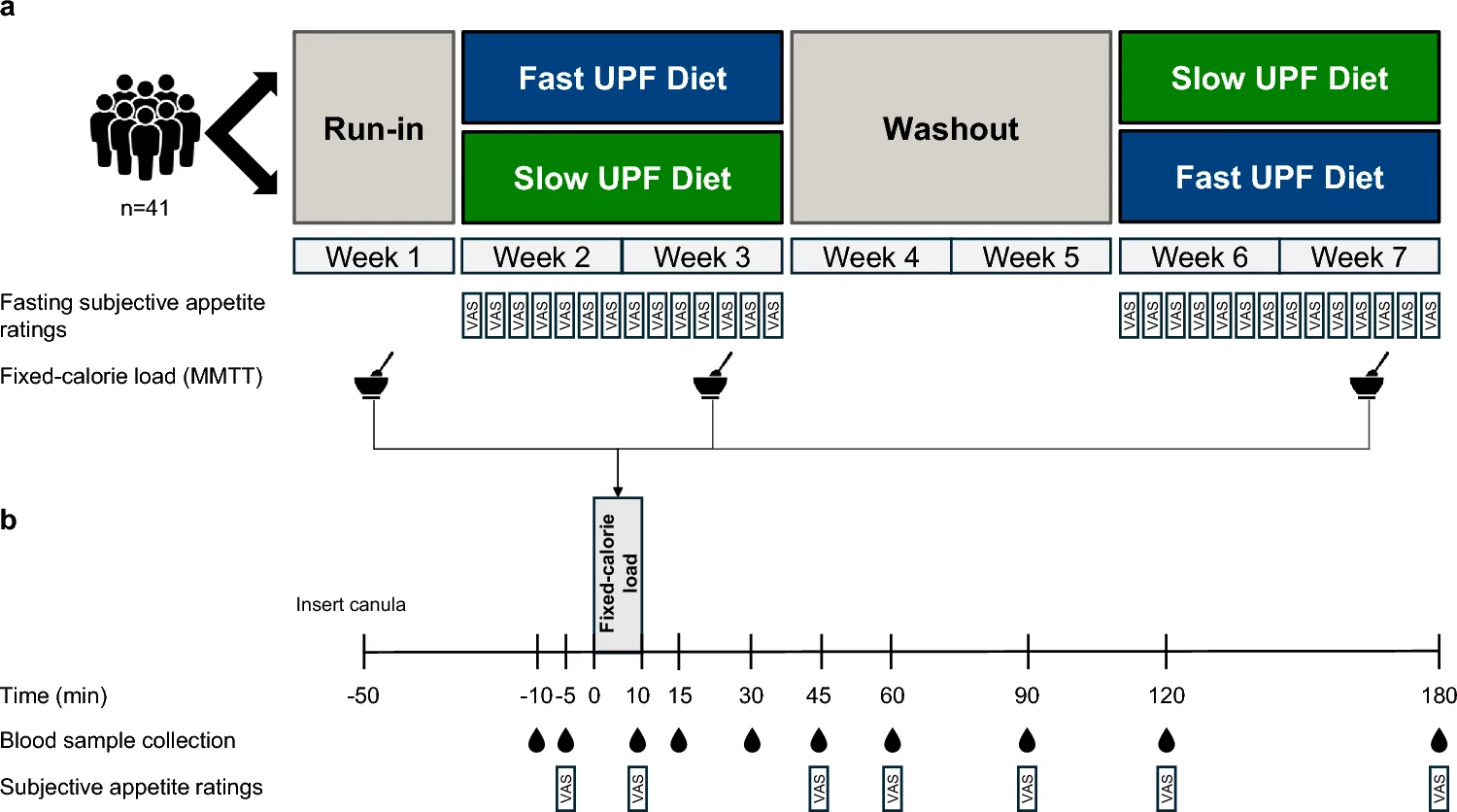
Overview of study design (**a**) and the metabolic challenge test with a fixed-calorie load (**b**). An intravenous cannula was placed 50 min prior to consumption of the fixed-calorie load (401 g, 552 kcal, 77 g carbohydrates, 15 g fat and 25 g protein). Blood samples were collected at 10 time points, from 10 min before to 180 min after consumption of the fixed-calorie load. Self-reported ratings of ‘hunger’, ‘fullness’, ‘thirst’, ‘desire to eat’ and ‘prospective consumption’ were assessed on a 100 mm VAS anchored from “not at all” to “extremely”, at 7 time points. Abbreviations: MMTT, mixed-meal tolerance test; UPF, ultra-processed food; VAS, visual analogue scale

This study represents the secondary outcomes from a randomized controlled diet intervention of which the primary outcome was to investigate the role of dietary eating rates on average daily energy intakes from UPFs. Trial protocol and the primary outcome are described in detail elsewhere [29, 30]. The study was preregistered at clinicaltrials.gov (NCT06113146), and the data analysis plan was preregistered at Open Science Framework (https://osf.io/afmkh). We aimed to assess whether satiety responsiveness changes following a 14-day period on a high-UPF diet. Satiety responsiveness was assessed (i) acutely through subjective and satiety hormone responses to a fixed-calorie load following each 14-day period of a diet high in UPFs (~ 95% EN% Nova 4) and (ii) over time; by monitoring changes in fasting-state satiety (pre- breakfast satiety ratings of hunger and fullness) across days on each UPF diet (faster and slower food textures). Participants with habitual low dietary intakes of UPFs (exclusion criteria: > 50% g/day Nova 4), completed two 14-day high-UPF diets. The UPF diets included a texture-derived slow eating rate diet (Slow UPF) and a texture-derived relative faster eating rate diet (Fast UPF), separated by a 14-day washout period during which participants returned to their habitual low-UPF diet (Fig. 1a). Prior to the first diet period, participants completed a 1-week run-in period during which they completed baseline measures and followed their habitual low-UPF diet. All participants completed both diet arms (within-subject) and three MMTTs were completed, one at baseline (run-in period) and on day 11 or 12 of each UPF-diet period. At each MMTT morning, the same fixed-calorie test meal was administered, after which participants’ rated their subjective appetite and had blood samples collected to assess acute satiety responsiveness following each 14-day high-UPF diet.

Written informed consent was obtained from all participants prior to the start of the study. The study was performed between January and November 2024 at the Health Research Unit of Wageningen University and Research in Wageningen, Netherlands and the study was approved by the Medical Ethical Committee ‘East-Netherlands’, Netherlands (ABR: NL83462.091.23). The study protocol complies with the Declaration of Helsinki for Medical Research involving Human Subjects.

### Participants

The study was powered based on the primary outcome of the RCT [30], which was between-diet difference in average daily energy intake (kcal/day) across 14 days (α = 0.05, power 1-β = 0.80, two-tailed) and at least 39 participants were needed to have an estimated effect size of 130 ± 280 kcal/day. A sensitivity analysis for the outcomes reported in the current manuscript (α = 0.05, power 1-β = 0.80) indicated that 34 participants were sufficient to detect a 10 ± 20% difference in subjective appetite ratings, corresponding to a moderate within-subject effect size (Cohen’s *d* of 0.5) [31]. This sample size is comparable to that of previous controlled feeding studies assessing subjective appetite and satiety hormone responses [32–34].

Participants (21–50 years, body mass index (BMI): 21–27 kg/m^2^) were recruited from Wageningen and surroundings through flyers, posters, and social media. Inclusion criteria included: healthy (self-report), normal appetite and commonly consuming three meals/day around the same times for at least five days a week (self-report). Exclusion criteria included: the use of chronic medication (other than contraceptives); smoking; food allergies or intolerances for any of the study foods; being a high restrained eater according to the Dutch Eating Behaviour Questionnaire (DEBQ) (cut-off for men > 2.89, for women: 3.39) [35]; body weight shift ≥ 5 kg within 6 months prior to participation; consuming more than 21 units/wk (men) or 14/units/wk (women) of alcohol; undertaking ≥ 4 h of moderate-to-vigorous physical activity per week; fasting haemoglobin values outside the range of 7.5–11.0 mmol/L for women and 8.5–11.0 mmol/L for men; and fasting blood glucose outside range: 3.5–8 mmol/L. Participants were excluded if > 50% of their total habitual food intake (grams) came from UPFs as determined by a newly developed food frequency questionnaire (UPFFQ). An overview of all in- and exclusion criteria is described in the study protocol [29].

### Dietary intake: baseline and study diets

Habitual dietary intake was estimated in the run-in week using a validated food diary application (Traqq) [36, 37] on two weekdays and one weekend day and participants were asked to record their food and beverage intakes on their smartphone.

The two UPF diets consisted of commercially available foods in the Netherlands classified as UPF by two independent coders using the Nova scheme [3], with 95% agreement between the two coders. The remaining 5% of products were classified by a third coder to reach consensus. When on the UPF diets, participants received all meals ad libitum across two (fast vs. slow) replicated 7-day menus. Participants consumed all of their main meals at the eating behaviour laboratory in the Health Research Unit at Wageningen University, except on weekends, when they received prepared and packaged meals and returned any leftovers after the weekend. Outside of the study foods, participants could drink water, coffee or tea without sugar or milk, and were asked to record their fluid intake away from the laboratory.

Texture-derived eating rate of the two UPF diets was intentionally designed to differ (Slow UPF, Fast UPF) in line with the primary outcome [38–41]. The UPF diets were matched on the level of the meal for portion size served (g), total energy served (kcal), variety, non-beverage energy density (kcal/gram), visual volume, liking and familiarity. On week-level the diets were matched closely on served macronutrient content (total and % energy), total fibre and salt, but priority was given to previously mentioned variables. Energy and nutrient intake were calculated based on packaging information and if unavailable, the Dutch food composition table was used (NEVO Table 2019, version 6). A detailed description of the two UPF diets and energy and macronutrients served and consumed on average *per* day can be found elsewhere [29, 30].

### Assessment of acute satiety responsiveness to a fixed-calorie load

At baseline and on day 11 or 12 of each diet period, a fixed-calorie load was administered during which subjective appetite ratings and blood samples were collected over a 180-min postprandial period to assess acute satiety responsiveness. Participants were instructed to avoid vigorous physical activity 24 h before the morning of each fixed-calorie test meal and were not allowed to eat or drink anything other than water after 20:00 pm the night before. Participants were allowed to have a glass of water (120 mL) in the morning before arrival.

Upon arrival at the research centre, an intravenous cannula was placed in an antecubital vein by a trained research nurse. Following a 50-min acclimatization period, two fasting blood samples were collected ten and five minutes before consumption of the fixed-calorie load. Further blood samples were collected at 10, 15, 30, 45, 60, 90, 120, and 180 min after consumption of the meal (Fig. 1). The cannula was removed after the final blood sample was collected.

To assess subjective appetite, participants rated their perceived ‘hunger’, ‘fullness’, ‘desire to eat’, ‘prospective consumption’ and ‘thirst’, on a 100 mm line scale anchored from “not at all” to “extremely” in an online questionnaire completed on a laptop, before consumption of the fixed-calorie load [42]. Participants repeated these ratings at 10 min (end of the meal), 45, 60, 90, 120 and 180 min after consumption of the fixed-calorie load (Fig. 1b).

The fixed-calorie test meal consisted of a UPF meal that was a fixed portion of a rice-based porridge (202 g) and 200 mL of chocolate milk (401 g, 552 kcal, 77 g CHO, 15 g fat, 25 g protein) [3]. Participants were given approximately 10 min to consume the full portion of the test meal and were instructed to drink the chocolate milk in one minute followed by the rice porridge which they had to consume within the remaining time, while eating in their normal way.

### Assessment of fasting-state appetite ratings over time during the UPF diets

To estimate changes in satiety responsiveness over time, participants rated their feelings of ‘hunger’, ‘fullness’, ‘desire to eat’ and ‘prospective consumption’ every morning before breakfast using the same line scale as described earlier.

### Blood sampling and laboratory analysis

Blood samples were collected in 3-mL EDTA-coated tubes and immediately iced after collection. Aprotinin and DPP-IV inhibitor were added to the collection tubes to prevent hormone degradation (Aprotinin from bovine lung, Hoffmann-La Roche AG and DPP-IV inhibitor, Merck). Blood samples were centrifuged at 1200 × g at 4 °C for 10 min and plasma was aliquoted and stored at − 80 °C until later analysis. Plasma GLP-1 (total), ghrelin (total), pancreatic polypeptide (PP) and PYY (total) were analysed using a multiplex assay (Meso Scale Discovery, Rockville, MD, USA). All samples from the same participant were analysed on the same assay to limit inter-assay effects and the detection range and inter- and intra-assay coefficients of variation are reported in Supplementary Table 1.

### Study outcomes

Acute satiety responsiveness was assessed at baseline and at the end of each UPF intervention arm for participants’ appetite ratings (hunger, fullness, desire to eat and prospective consumption) and for timepoint changes across their fasting (T-10 and T-5) and postprandial plasma concentrations of ghrelin, GLP-1, PYY and PP. Changes in fasting-state subjective satiety (pre- breakfast appetite ratings of hunger and fullness) were assessed across days within each UPF diet.

### Allocation of diet order

Participants were randomly allocated in a 1:1 ratio to one of the two diet orders (Fast UPF > Slow UPF or Slow UPF > Fast UPF) using block-stratified randomization envelopes by two researchers responsible for study coordination. All participants completed the baseline calorie load test during the run-in period while adhering to their habitual diet. The order of the post-UPF diet calorie load tests depended on the diet order assigned to each participant. Participants were blinded to the true study aim and were debriefed upon completion of the study. Randomization and concealment of the study aim are described in detail elsewhere [30].

### Statistical analysis

All statistical analyses were performed using SAS (version 9.4; SAS Institute Inc., Cary, NC, USA) and *p*-values ≤ 0.05 were considered statistically significant. All analyses were conducted on an intention-to-treat basis, including data from the two participants who dropped out during the run-in week and first intervention week of the study as pre-registered (https://osf.io/afmkh). Descriptive statistics are presented as means and 95% confidence intervals (95% CIs), unless otherwise stated. Normality of the data was visually inspected using histograms and QQ-plots. Plasma GLP-1, ghrelin, PP, and PYY concentrations were not normally distributed and therefore log-transformed (ln, natural logarithm) before analysis. Log-transformed GLP-1, ghrelin, PP, and PYY were normally distributed. For these variables geometric means and back-transformed 95% CIs are reported, unless otherwise stated. Primary outcomes were tested for an effect of diet order, and no order effects were found.

A repeated-measures (within-subject) mixed model (PROC MIXED) with covariance structure AR(1) was used to test for a main effect of study visit on post-meal appetite ratings to the fixed-calorie load. The model included Diet (Baseline (run-in period), post-Fast UPF and post-Slow UPF) and timepoint (min) and their interaction as fixed factors. Participant, block and diet order were added as random variables. The covariance structure autoregressive (AR(1)) was selected based on the log-likelihood difference. The same model was used to test for a main effect of study visit on postprandial plasma concentrations of GLP-1, ghrelin, PP, and PYY. When main or interaction effects were significant, post-hoc Tukey corrected t-tests were conducted to assess whether differences between diet arms were significant.

Plasma GLP-1, ghrelin, PP, and PYY concentrations measured at ten and five minutes before meals (T = − 10 and T = − 5) were averaged to define fasting levels. Total areas under the curve (AUCs) were calculated for ratings of ‘Hunger’, ‘Fullness’, ‘Desire to eat’, ‘Prospective consumption’, ‘Thirst’, and plasma concentrations of GLP-1, ghrelin, PP, and PYY [43]. Differences in pre-calorie load appetite ratings, fasting plasma concentrations, total AUC values were analysed using a repeated-measures (within-subject) mixed model (PROC MIXED) with covariance structure CS, where study visit was added as a fixed factor and participant, block and diet order as random factors. If significant, post-hoc Tukey tests were conducted to assess whether difference between arms were significant.

A repeated-measures (within-subject) mixed model (PROC MIXED) with covariance structure AR(1) was used to assess changes in fasting-state appetite ratings over time during the UPF diets. The model included diet (Fast UPF, Slow UPF) and day and their interaction as fixed factors. Participant, block and diet order were added as random variables. When main or interaction effects were significant, post-hoc Tukey corrected t-tests were conducted to assess whether differences between diet arms were significant.

## Results

### Participant characteristics

**Fig. 2:**
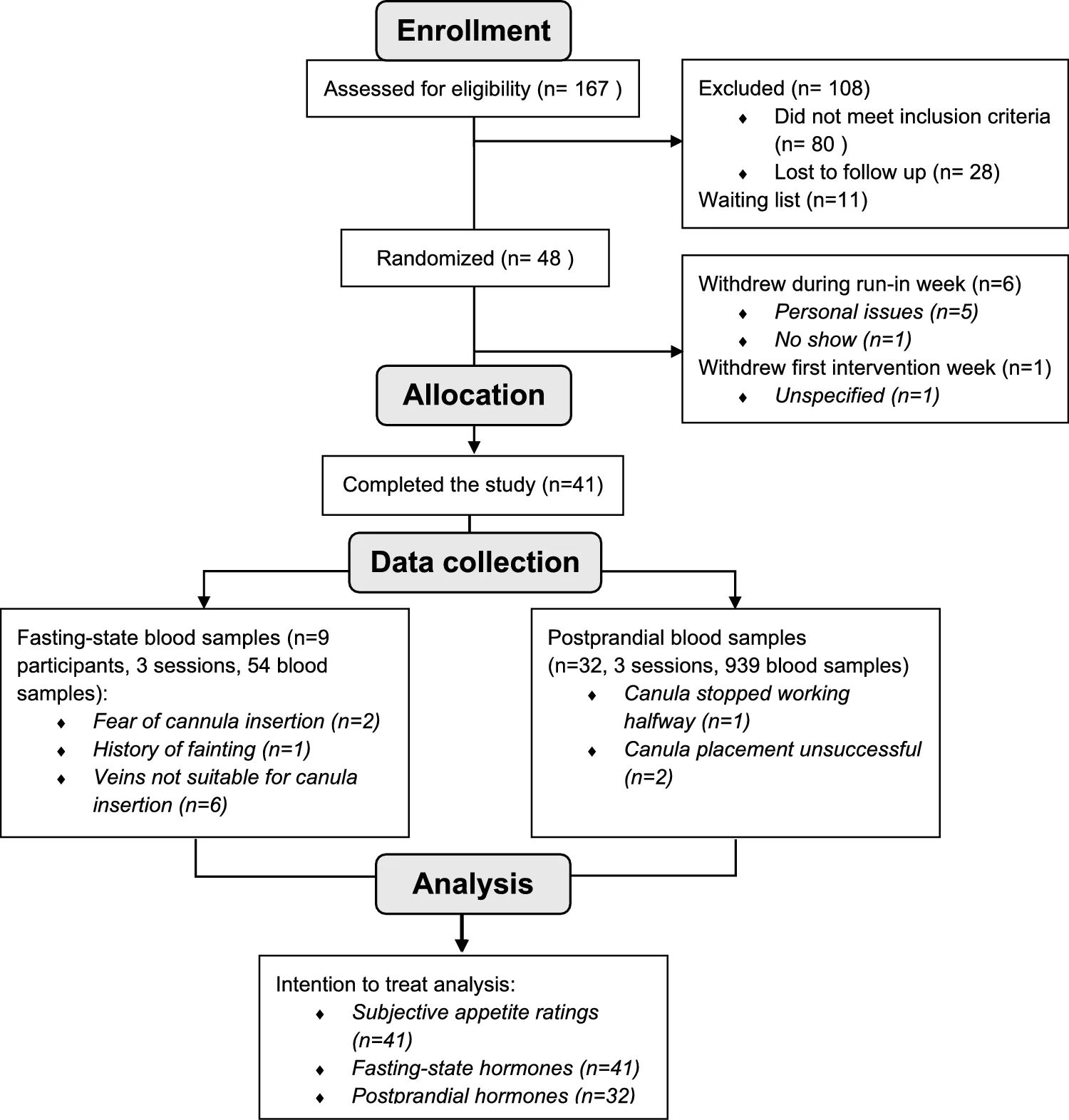
CONSORT flow diagram

In total 167 participants joined the information session of which 48 participants were eligible for participation. Six participants dropped out during the run-in period, and one participant dropped out during the first week of the first intervention period. In total 41 participants (n = 21 male; mean ± SD: age 27 ± 5 y, BMI 23.4 ± 1.9 kg/m^2^) completed the study and were included for data analysis (Fig. 2). Fasting hormone concentrations and subjective appetite ratings are reported for the total cohort. Nine participants were not cannulated, and no postprandial blood samples were collected due to either fear of cannulation, a history of fainting or unsuitable veins. Satiety hormone responses are therefore reported for 32 participants. These 32 participants were representative of the total cohort (n = 41) and were on average 27 ± 2 years old with a BMI of 23.6 ± 2.3 kg/m^2^ (Table 1).

**Table 1:** Baseline characteristics of the study participants

	Total (n = 41)			Sub-sample (cannulated, n = 32)		
	All (n = 41)	Male (n = 21)	Female (n = 20)	All (n = 32)	Male (n = 17)	Female (n = 15)
Age at baseline (y)	27 ± 5	27 ± 6	27 ± 5	27 ± 2	28 ± 6	27 ± 6
Body weight (kg)	70 ± 10	75 ± 10	64 ± 6	71 ± 11	77 ± 10	64 ± 6
FFM (kg)	52 ± 11	60 ± 8	43 ± 4	53 ± 11	61 ± 9	44 ± 5
FM (kg)	18 ± 6	15 ± 4	21 ± 5	17 ± 6	15 ± 5	20 ± 5
Height (cm)	173 ± 9	179 ± 7	166 ± 5	173 ± 10	179 ± 8	166 ± 6
BMI (kg/m^2^)	23.4 ± 2.1	23.5 ± 2.4	23.4 ± 1.9	23.6 ± 2.3	23.8 ± 2.5	23.4 ± 2.0
HbA1c (mmol/mol)^a^	32 ± 3	32 ± 4	32 ± 2	32 ± 3	31 ± 4	32 ± 2
Systolic blood pressure (mmHg)	115 ± 13	122 ± 11	107 ± 10	117 ± 12	123 ± 11	110 ± 10
Diastolic Blood pressure (mmHg)	67 ± 6	66 ± 6	67 ± 7	67 ± 6	67 ± 6	68 ± 6
Dietary restraint score^b^	2.0 ± 0.5	1.9 ± 0.5	2.1 ± 0.6	1.9 ± 0.6	1.8 ± 0.5	2.0 ± 0.7
Habitual UPF intake (% g/day)	21 ± 14	20 ± 14	22 ± 13	21 ± 14	20 ± 15	22 ± 13
Habitual UPF intake (EN%)	45 ± 22	41 ± 23	49 ± 20	45 ± 22	41 ± 23	49 ± 21

Data are shown for all participants (n = 41) for subjective satiety ratings and fasting hormone concentrations, and for the sub-sample of cannulated participants (n = 32) that provided additional postprandial blood samples. Values are presented as mean ± SD

^a^Due to missing data, HbA1c is mean of n = 39, n = 19 male total study sample, n = 15 male cannulated study sub-sample

^b^Measured with the Dutch Eating Behaviour Questionnaire (DEBQ): cut-off for men > 2.89, cut-off for women > 3.39 [35]

Abbreviations: BMI, Body mass index; EN, Energy; FM, Fat mass; FFM, Fat free mass

### Diet effect on energy intake

These outcomes are reported in detail elsewhere [30]. Estimated habitual daily energy intakes at baseline was 2,044 kcal/day, of which 973 kcal (45%) came from UPFs. During the UPF diets, participants consumed on average 2301 kcal/day on the Slow UPF diet (98% EN% Nova 4) and 2671 kcal/day on the Fast UPF diet (94% EN% Nova 4) (Supplementary Table 2).

### Acute subjective satiety responses to fixed-calorie load following high-UPF diets

**Fig. 3:**
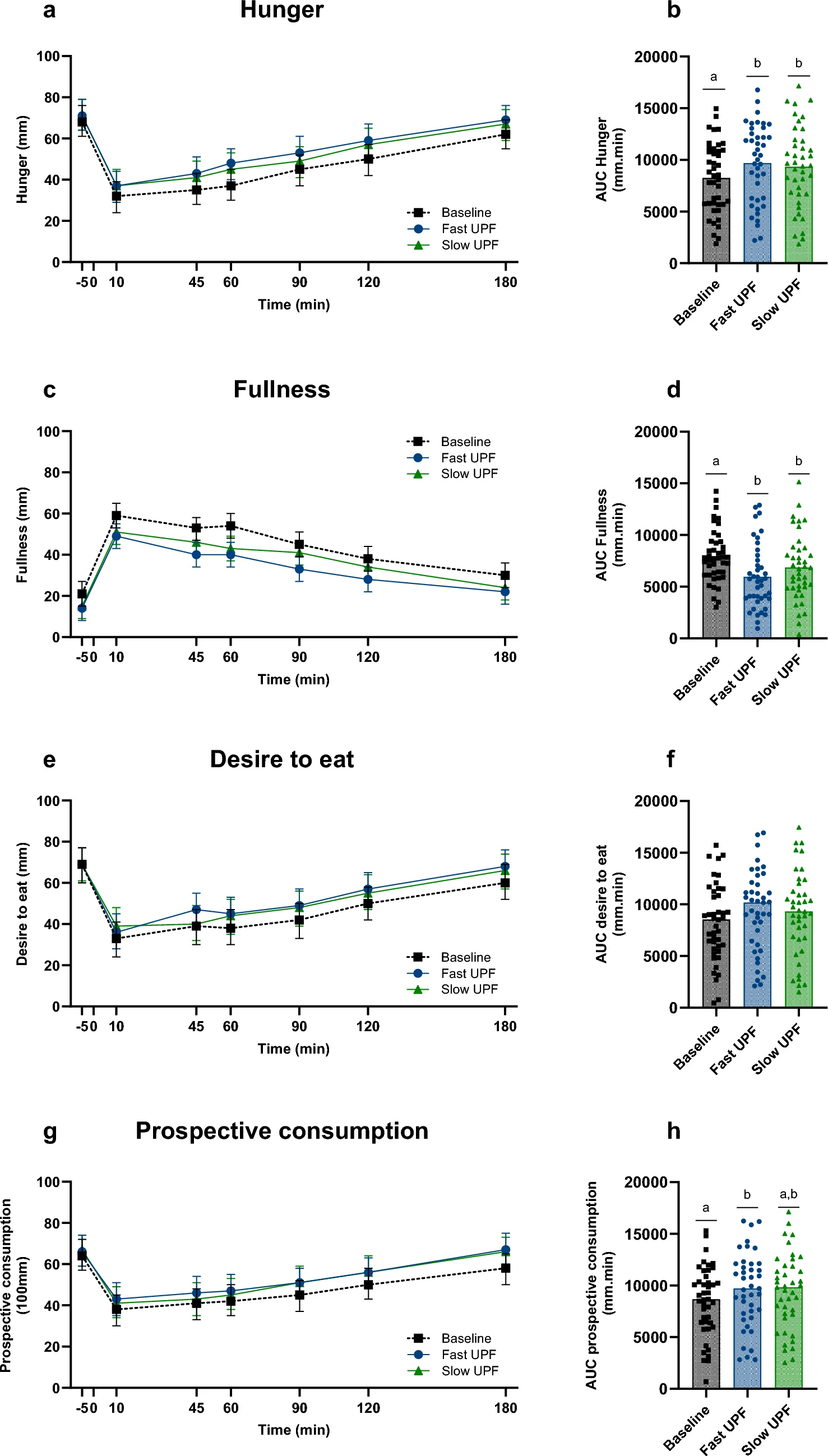
Ratings of Hunger (**a**), Fullness (**c**), Desire to eat (**e**) and Prospective consumption (**g**) over 180 min following consumption of the fixed-calorie load and total AUC for Hunger (**b**), Fullness (**d**), Desire to eat (**f**) and Prospective consumption (**h**). Values are mean (95% CI), and *p*-values are derived from repeated-measures linear mixed models with post-hoc Tukey corrected two-sided t-tests with significance set at *p* ≤ 0.05. The diet x time interactions were as follows: Hunger **a**: F(12,812) = 0.5, *p* = 0.941; Fullness **c**: F(12,812) = 0.8, *p* = 0.669; Desire to eat **e**: F(12,812) = 0.8, *p* = 0.687; Prospective consumption **g**: F(12,812) = 0.4, *p* = 0.974. Total AUCs were compared between treatments and the Diet effect was as follows: Hunger **b**: F(2,80) = 6.7, *p* = 0.002; Fullness **d**: F(2,80) = 11.2, *p* < 0.0001; Desire to eat **f**: F(2,80) = 3.1, *p* = 0.051; Prospective Consumption **h**: F(2,80) = 3.5, *p* = 0.036. AUC values without a common letter differ *p* ≤ 0.05 with Tukey adjustments for multiple comparisons. Abbreviations: AUC, Area under the curve; CI, Confidence interval; UPF, ultra-processed food

Overall the fasting-state appetite ratings were similar (Δ 0–6 mm on 100 mm VAS) across baseline and following the UPF diets (*p* > 0.156). Fullness ratings differed (Δ 7 mm on 100 mm VAS, *p* = 0.032) but this can be considered negligible as it is smaller than 10 mm (100 mm scale) (Table 2) [44]. Post-calorie load subjective appetite ratings per individual timepoint were similar at baseline and following the UPF diets (Fig. 3) and diet x time interactions were not significant for ratings of hunger, fullness, desire to eat or prospective consumption (all, *p* > 0.05). Although no differences were observed at individual timepoints, analysis of total AUCs revealed higher hunger (F(2,80) = 6.7, *p* = 0.002) and lower fullness (F(2,80) = 11.2, *p* < 0.0001) following both UPF diets compared to baseline (Fig. 3). Total AUCs for desire to eat (F(2,80) = 3.1, *p* = 0.051) and thirst (F(2,80) = 0.8, *p* = 0.456) were similar.

**Table 2:** Fasting-state appetite ratings and fasting-state hormone concentrations (n = 41) at baseline and following the Fast and Slow UPF diets

	Baseline	Fast UPF diet	Slow UPF diet	*p*-value
*Subjective appetite ratings*				
Fasting hunger (mm)	68 (62–75)	71 (65–78)	71 (65–78)	0.538
Fasting fullness (mm)	21 (16–25)^a^	14 (9–19)^b^	15 (11–20)^a,b^	0.032
Fasting desire to eat (mm)	69 (62–76)	69 (61–76)	69 (62–76)	0.992
Fasting prospective consumption (mm)	64 (59–70)	66 (61–72)	67 (61–72)	0.724
Fasting thirst (mm)	67 (59–74)	61 (53–69)	63 (55–71)	0.156
*Satiety hormones*				
Fasting GLP-1 (pmol/L)	6.0 (5.3–6.7)	6.8 (6.0–7.7)	6.6 (5.8–7.5)	0.058
Fasting ghrelin (pg/mL)	96 (72–129)	100 (75–134)	99 (74–133)	0.569
Fasting PP (pg/mL)	52 (41–66)^a^	64 (50–81)^a^	64 (51–82)^a^	0.032
Fasting PYY (pg/mL)	62 (49–79)^a^	73 (57–92)^b^	68 (53–86)^a,b^	0.029

Appetite ratings are presented as mean (95% CI), and fasting-state hormone concentrations are presented as geometric means and back-transformed 95% CIs. Means were derived from repeated-measures linear mixed models, with *p* ≤ 0.05 considered statistically significant. Means with different superscript letters differ significantly as indicated by Tukey-adjusted post hoc tests

Abbreviations: CI, Confidence interval; GLP-1, Glucagon-like peptide-1; PP, Pancreatic polypeptide; PYY, Peptide YY; UPF, ultra-processed food

There was no effect of eating rate of the UPF diets on acute satiety responsiveness, as fasting-state and postprandial subjective appetite ratings to the fixed-calorie load were similar after the Fast and Slow UPF diets (all, *p* > 0.05).

### Acute satiety hormone responses to fixed-calorie load following high-UPF diets

Fasting-state plasma hormone concentrations are presented in Table 2. Fasting PYY concentrations were 17% higher following the Fast UPF diet compared to when measured at baseline (GMR = 1.17, 95% CI 1.04–1.31, *p* = 0.022).

**Fig. 4:**
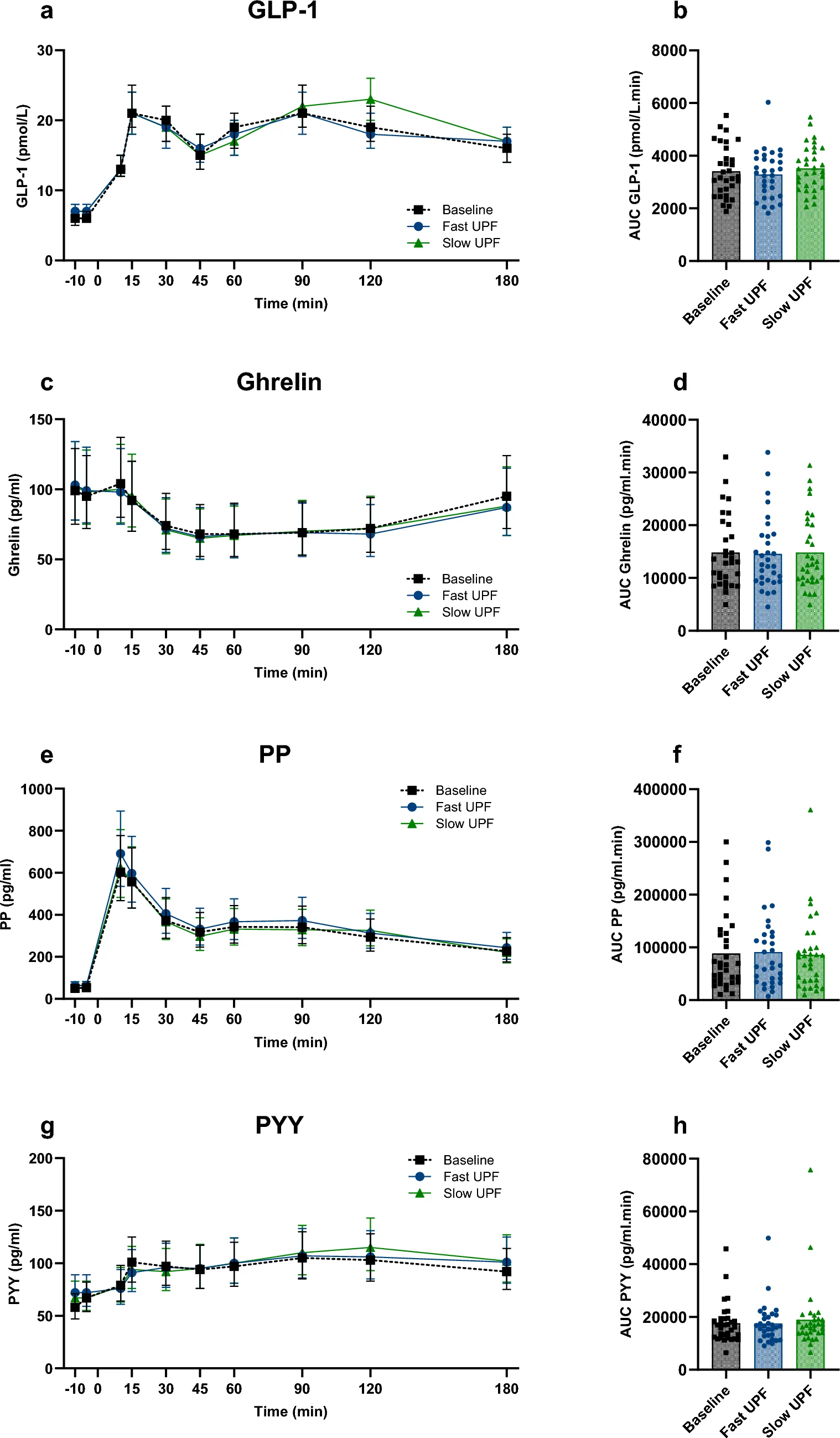
Plasma GLP-1 (**a**), Ghrelin (**c**), PP (**e**), PYY (**g**) responses to the fixed-calorie load for baseline and the Fast and Slow UPF diets. Data are presented as geometric mean (back-transformed 95% CI) and *p*-values are derived from repeated-measures linear mixed models with post-hoc Tukey corrected two-sided t-tests with significance set at *p* ≤ 0.05. The diet x time interactions were as follows: GLP-1 **a**: F(18,945) = 1.0, *p* = 0.427; Ghrelin **c**: F(18,944) = 0.6, *p* = 0.869; PP **e**: F(18,945) = 0.7, *p* = 0.847; PYY **g**: F(18,945) = 1.6, *p* = 0.052. Total AUC values for GLP-1 (**b**), Ghrelin (**d**), PP (**f**) and PYY (**h**) concentrations were compared between treatments and main diet effects were: GLP-1 **b**: F(2,59) = 1.2, *p* = 0.304; Ghrelin **d**: F(2,59) = 0.2, *p* = 0.848; PP **f**: F(2,59) = 1.8, *p* = 0.176; and PYY **h**: F(2,59) = 1.4, *p* = 0.266. Abbreviations: AUC, Area under the curve; CI, Confidence interval; GLP-1, glucagon-like peptide-1; PP, pancreatic polypeptide; PYY, Peptide YY; UPF, ultra-processed food

Postprandial satiety hormone responses to the fixed-calorie load were similar at baseline and following each UPF diet, with no significant diet x time interactions observed for GLP-1 (F(18,945) = 1.0, *p* = 0.427), ghrelin (F(18,944) = 0.6, *p* = 0.869), PP (F(18,945) = 0.7, *p* = 0.847) and PYY (F(18,945) = 1.6, *p* = 0.052) (Fig. 4). Consistent with this, no main diet effects were observed for total AUCs of GLP-1: F(2,59) = 1.2, *p* = 0.304), ghrelin: (F(2,59) = 0.2, *p* = 0.848), PP: (F(2,59) = 1.8, *p* = 0.176), and PYY: (F(2,59) = 1.4, *p* = 0.266) for the fixed-calorie load, with comparable responses at baseline and post-UPF diets (Fig. 4).

Eating rate of UPF diets (Slow UPF vs Fast UPF) did not significantly influence fasting-state or postprandial satiety hormone concentrations to the fixed-calorie load test-meal (all, *p* > 0.05).

### Over-time fasting-state subjective appetite ratings

**Fig. 5:**
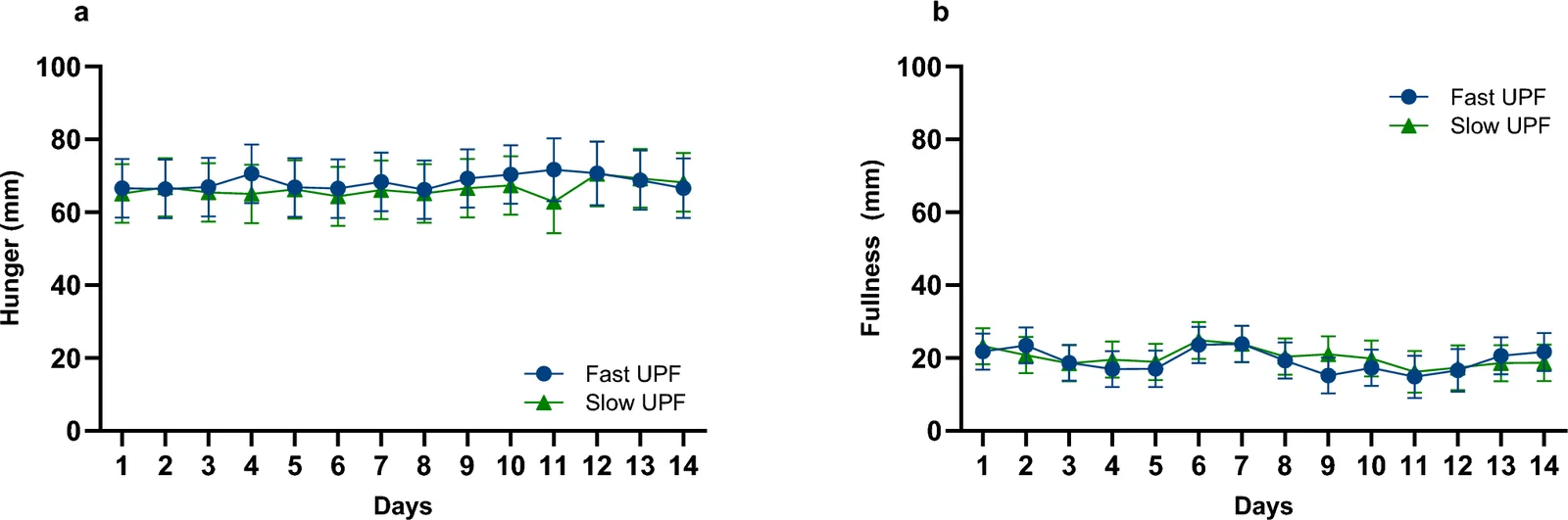
Fasting-state (pre-breakfast) Hunger (**a**) and Fullness (**b**) ratings measured daily during each UPF diet intervention period. Values are mean (95% CI), and *p*-values are derived from repeated-measures linear mixed models with post-hoc Tukey corrected two-sided t-tests with significance set at *p* ≤ 0.05. The diet x time interactions were as follows: Hunger **a**: F(13,977) = 0.9, *p* = 0.586; and Fullness **b**: F(13,977) = 1.0, *p* = 0.442. Abbreviations: CI, Confidence interval; UPF, ultra-processed food

Fasting-state appetite ratings did not differ between the UPF diets over the course of the intervention period (all, <10 mm) (Fig. 5) [44]. There were no significant diet x time interaction effects observed for fasting-state ratings of hunger (F(13,977) = 0.9, *p* = 0.586) and fullness (F(13,977) = 1.0, *p* = 0.442) or fasting-state ratings of desire to eat (diet x day interaction effect: F(13,977) = 0.4, *p* = 0.971) and prospective consumption (diet x day interaction effect: F(13,977) = 0.7, *p* = 0.733).

## Discussion

We examined whether transitioning from a habitual low-UPF diet to a diet high in UPFs (~ 95%EN) affects satiety responsiveness as measured by both acute and ‘over-time’ satiety responses of participants, and whether these responses were moderated by eating rate of the diet. Results show no clear systematic evidence of an adaptation in acute satiety responses (i.e. time-series subjective appetite ratings and satiety hormone responses) to a fixed-calorie load following each UPF diet period, though there was a small and significant change in AUC for hunger and fullness following each UPF diet. We further did not observe an ‘over-time’ adaptation of subjective feelings of appetite (i.e. morning hunger ratings) during each of the high-UPF diet intervention arms. Similarly, the eating rate of each UPF diet did not influence participants satiety responsiveness (acute or over-time responses).

For the acute responses following the high-UPF diets, we observed significant differences in the total area under the curve (AUC) for subjective ratings of ‘hunger’ and ‘fullness’ when comparing the two high-UPF diets to the same responses at baseline (Fig. 3). These differences were small but significant [44], and we speculate this may have arisen due to the smaller fixed portion nature of the fixed-calorie load, which was in contrast to the wider ad libitum nature of the previous 14-day period on a UPF diet. Memory for recent eating episodes can help shape expectations of satiety [45, 46], and memory of larger portion sizes has been associated with greater expected satiety compared to smaller portions [47]. All meals during the UPF diets were served ad libitum (approximately 6000 kcals/day or three times a normal portion size), in line with the primary outcome of the study, which was to estimate the average daily energy intake on a Fast UPF compared to a Slow UPF diet [30]. The current study reports secondary analyses from the Restructure randomized controlled trial and was designed for this primary outcome, rather than to detect acute satiety responsiveness to a fixed-calorie load following UPF diets. As such the sample size for the satiety comparison may have been relatively large for these secondary outcomes [44]. Subjective satiety ratings were variable and although AUC differed significantly, there were no differences at individual postprandial timepoints, or observed concurrent changes in neuro-endocrine response to the fixed-calorie meal.

A limitation of the current comparison is that the baseline measures of satiety were based on a standard 7-day free living diet, where a more appropriate comparator would have been a controlled 14-day run-in period on the baseline diet (≤  50% UPF), to have an equivalent level of intervention [48]. For participants, completing a diet intervention in a semi-residential controlled feeding context may also have affected subjective appetite ratings, as awareness of dietary restrictions and intake monitoring could influence outcomes even when participants were blinded to the true aim of the trial [49]. The change in total AUC trends for ‘hunger’ and ‘fullness’ merit further consideration in light of putative mechanisms of UPF effects on intake, but should also be interpreted with caution. The current exploratory comparison does not suggest a strong or systematic effect of high-UPF diets on satiety responsiveness following nutrient matched ad libitum diets. Fasting hunger and fullness ratings assessed each morning did not change over time during the UPF intervention periods, and remained stable across both 14-day high-UPF diets. Differences in satiety responsiveness as captured in the AUC comparisons did not affect later food intake behaviour and participants did not consume beyond their energy requirements, as they maintained body weight on the Fast UPF arm, and lost fat mass on the Slow UPF diet [30]. Future research is needed to confirm these findings, but our preliminary comparison study does not suggest a dysregulation of satiety responses following a period of high-UPF exposure, nor that diets high in UPFs promote a stronger appetitive response or stimulate hunger and additional calorie intakes over time.

Acute satiety hormone responses were similar following the UPF diets, except for observed higher fasting PYY levels following the Fast UPF diet relative to baseline. This contrasts with a previous study reporting *lower* fasting PYY after a 14-day UPF diet compared with an unprocessed diet [50]. However, the higher fasting PYY observed in our study was not observed when comparing differences in postprandial hormone responses, as PYY but also GLP-1, ghrelin, and PP responses for the fixed-calorie load remained unchanged following both UPF diet interventions. Studying postprandial responses across a series of replicated fixed-calorie load meal tests (MMTTs) is a strength of the current study, as it provides greater insights into appetite regulation than fasted measures, by capturing the dynamic rather than static regulation of satiety hormones in response to food intake [51]. Our results are consistent with previous acute feeding trials investigating satiety responses to single UPF meals [33, 52]. These studies reported no differences in postprandial ghrelin, or GLP-1 responses following a UPF breakfast compared with processed [33] or non-UPF breakfasts [52], suggesting no acute effect of consumption of foods classified as UPF on satiety hormone responses. The overall absence of differences in the satiety hormones, with the exception of fasting PYY, suggests that in the current study prolonged UPF exposure did not substantially alter satiety hormone responses. Previous research has shown that satiety signalling can be disrupted by environmental endocrine disruptors, such as micro-plastics and pesticides that act on the hypothalamus to impair appetite regulation and energy balance [53, 54]. However, results of current the study suggest that satiety signalling is not affected when nutrient matched high-UPF diets are adopted for 14-days, using widely commercially available food products.

Circulating satiety hormone levels are sensitive to changes in energy balance, body composition, and metabolic status [55–57]. Diet induced weight loss via caloric restriction can result in acute compensatory changes in appetite-related gastrointestinal hormone responses that favour weight gain [58, 59], which can even persist one year after initial weight loss [60]. The intervention duration (14-days) and intensity (*ad-libitum* access to foods) in the current trial was focused on changes in daily energy intake rather than weight loss, yet participants lost on average 0.43 kg of fat mass on the Slow-UPF diet, reflecting a cumulative reduction of over 5200 kcal in energy intake [30]. Despite this modest change in body composition, circulating satiety hormones following the fixed-calorie load remained unchanged, indicating sustained reductions in texture-derived eating rate did not elicit compensatory physiological adaptations in circulating satiety hormones. These preliminary findings merit further consideration and suggest that using food-based approaches to reduce eating speed may support sustained reductions in both energy intake and potentially fat mass, without altering circulating satiety hormone levels among normal-weight individuals.

Satiety responsiveness was not affected by differences in texture-derived eating rate between the two UPF diets. Both acute and ‘over-time’ satiety responsiveness did not differ following the Fast and Slow UPF diets. Recent research comparing satiety responses to lunch meals differing in texture-based eating rate (slow vs fast) and degree of processing (minimally processed vs ultra-processed) has shown that slower eating rate for the UPF meal promoted a higher post-meal satiety response. Though this effect was not found for the slowly consumed minimally processed meal [61]. Taken together, these findings suggest that eating behaviour, rather than degree of food processing, is likely more influential in driving differences in postprandial fullness, although it is worth noting that the magnitude of the observed effects in all cases were relatively small. While some have suggested public health policy should focus on reducing UPF intake entirely [62], our findings suggest that identifying the food properties that influence energy intake, satiety and energy balance independently of degree of food processing, may provide more targeted and effective opportunities to reformulate and improve the food environment, and reduce the risk of caloric overconsumption [63].

## Conclusion

Our results show that transitioning from a diet low in UPFs to a diet high in UPFs has no clear and systematic effect on satiety responsiveness. We observed small but significant differences in summary measures of acute satiety responsiveness (AUC hunger and fullness) but no change in time-series postprandial subjective satiety responses to a replicated fixed-calorie load meal following exposure to a high-UPF diet. There were no significant changes in satiety hormone responses to the fixed-calorie meal, and over-time satiety responsiveness remained stable across two 14-day high-UPF diet periods. The eating rate of the UPF diets did not influence either subjective or hormonal satiety response to the fixed-calorie meal. Our findings suggest that doubling dietary intakes of foods classified as UPFs to ~95%EN across two 14-day UPF diet interventions did not systematically alter acute satiety or fasting-state satiety responses. Future research should aim to confirm whether these trends remain across different dietary contexts and populations, to independently replicate the comparison of the effect of UPFs on satiety responsiveness when exploring mechanisms proposed to moderate energy intake from UPFs.

## Supplementary Information

Below is the link to the electronic supplementary material.

Supplementary file1 (DOCX 19 kb)

## Data Availability

No datasets were generated or analysed during the current study.

## Abbreviations

AUC: Area under the curve
BMI: Body mass index
CI: Confidence interval
GLP-1: Glucagon-like peptide-1
DEBQ: Dutch eating behaviour questionnaire
MMTT: Mixed-meal tolerance test
PP: Pancreatic polypeptide
PYY: Peptide tyrosine tyrosine
RCT: Randomized controlled trial
UPF: Ultra-processed food
VAS: Visual analogue scale

## References

[1] Ulug E, Acikgoz Pinar A, Yildiz BO (2025) Impact of ultra-processed foods on hedonic and homeostatic appetite regulation: a systematic review. Appetite 213:108139. 10.1016/j.appet.2025.108139 40388988

[2] Askari M, Heshmati J, Shahinfar H, Tripathi N, Daneshzad E (2020) Ultra-processed food and the risk of overweight and obesity: a systematic review and meta-analysis of observational studies. Int J Obes (Lond) 44(10):2080–2091. 10.1038/s41366-020-00650-z 32796919

[3] Monteiro CA, Cannon G, Levy RB, Moubarac JC, Louzada ML, Rauber F et al (2019) Ultra-processed foods: what they are and how to identify them. Public Health Nutr 22(5):936–941. 10.1017/S1368980018003762 PMC10260459 30744710

[4] Fardet A (2016) Minimally processed foods are more satiating and less hyperglycemic than ultra-processed foods: a preliminary study with 98 ready-to-eat foods. Food Funct 7(5):2338–2346. 10.1039/c6fo00107f 27125637

[5] Valicente VM, Peng CH, Pacheco KN, Lin L, Kielb EI, Dawoodani E et al (2023) Ultraprocessed foods and obesity risk: a critical review of reported mechanisms. Adv Nutr 14(4):718–738. 10.1016/j.advnut.2023.04.006 PMC10334162 37080461

[6] Juul F, Martinez-Steele E, Parekh N, Monteiro CA (2025) The role of ultra-processed food in obesity. Nat Rev Endocrinol 21(11):672–685. 10.1038/s41574-025-01143-7 40659796

[7] Juul F, Vaidean G, Parekh N (2021) Ultra-processed foods and cardiovascular diseases: potential mechanisms of action. Adv Nutr 12(5):1673–1680. 10.1093/advances/nmab049 PMC8483964 33942057

[8] Srour B, Kordahi MC, Bonazzi E, Deschasaux-Tanguy M, Touvier M, Chassaing B (2022) Ultra-processed foods and human health: from epidemiological evidence to mechanistic insights. Lancet Gastroenterol Hepatol 7(12):1128–1140. 10.1016/S2468-1253(22)00169-8 35952706

[9] Dupont D, Le Feunteun S, Marze S, Souchon I (2018) Structuring food to control its disintegration in the gastrointestinal tract and optimize nutrient bioavailability. Innov Food Sci Emerg Technol 46:83–90. 10.1016/j.ifset.2017.10.005

[10] Abiega-Franyutti P, Freyre-Fonseca V (2021) Chronic consumption of food-additives lead to changes via microbiota gut-brain axis. Toxicology 464:153001. 10.1016/j.tox.2021.153001 34710536

[11] Simmons AL, Schlezinger JJ, Corkey BE (2014) What are we putting in our food that is making us fat? Food additives, contaminants, and other putative contributors to obesity. Curr Obes Rep 3(2):273–285. 10.1007/s13679-014-0094-y PMC4101898 25045594

[12] Song Z, Song R, Liu Y, Wu Z, Zhang X (2023) Effects of ultra-processed foods on the microbiota-gut-brain axis: the bread-and-butter issue. Food Res Int 167:112730. 10.1016/j.foodres.2023.112730 37087282

[13] Small DM, DiFeliceantonio AG (2019) Processed foods and food reward. Science 363(6425):346–347. 10.1126/science.aav0556 30679360

[14] Kelly AL, Baugh ME, Oster ME, DiFeliceantonio AG (2022) The impact of caloric availability on eating behavior and ultra-processed food reward. Appetite 178:106274. 10.1016/j.appet.2022.106274 PMC9749763 35963586

[15] Forde CG, Mars M, de Graaf K (2020) Ultra-processing or oral processing? A role for energy density and eating rate in moderating energy intake from processed foods. Curr Dev Nutr 4(3):nzaa019. 10.1093/cdn/nzaa019 PMC7042610 32110771

[16] Rolls BJ, Cunningham PM, Diktas HE (2020) Properties of ultraprocessed foods that can drive excess intake. Nutr Today. 10.1097/NT.0000000000000410

[17] Benelam B (2009) Satiation, satiety and their effects on eating behaviour. Nutr Bull 34(2):126–173. 10.1111/j.1467-3010.2009.01753.x

[18] Dhillo WS (2007) Appetite regulation: an overview. Thyroid 17(5):433–445. 10.1089/thy.2007.0018 17542673

[19] Cummings DE, Overduin J (2007) Gastrointestinal regulation of food intake. J Clin Invest 117(1):13–23. 10.1172/JCI30227 PMC1716217 17200702

[20] Forde CG, de Graaf K (2022) Influence of sensory properties in moderating eating behaviors and food intake. Front Nutr 9:841444. 10.3389/fnut.2022.841444 PMC8899294 35265658

[21] Kokkinos A, le Roux CW, Alexiadou K, Tentolouris N, Vincent RP, Kyriaki D et al (2010) Eating slowly increases the postprandial response of the anorexigenic gut hormones, peptide YY and glucagon-like peptide-1. J Clin Endocrinol Metab 95(1):333–337. 10.1210/jc.2009-1018 19875483

[22] Zhu Y, Hsu WH, Hollis JH (2013) Increasing the number of masticatory cycles is associated with reduced appetite and altered postprandial plasma concentrations of gut hormones, insulin and glucose. Br J Nutr 110(2):384–390. 10.1017/S0007114512005053 23181989

[23] Goh AT, Choy JYM, Chua XH, Ponnalagu S, Khoo CM, Whitton C et al (2021) Increased oral processing and a slower eating rate increase glycaemic, insulin and satiety responses to a mixed meal tolerance test. Eur J Nutr 60(5):2719–2733. 10.1007/s00394-020-02466-z 33389082

[24] Hawton K, Ferriday D, Rogers P, Toner P, Brooks J, Holly J et al (2019) Slow down: behavioural and physiological effects of reducing eating rate. Nutrients 11(1):50. 10.3390/nu11010050 PMC6357517 30591684

[25] Karl JP, Young AJ, Rood JC, Montain SJ (2013) Independent and combined effects of eating rate and energy density on energy intake, appetite, and gut hormones. Obesity 21(3):E244–E252. 10.1002/oby.20075 23592679

[26] Robinson E, Almiron-Roig E, Rutters F, de Graaf C, Forde CG, Tudur Smith C et al (2014) A systematic review and meta-analysis examining the effect of eating rate on energy intake and hunger. Am J Clin Nutr 100(1):123–151. 10.3945/ajcn.113.081745 24847856

[27] Ferriday D, Bosworth ML, Godinot N, Martin N, Forde CG, Van Den Heuvel E et al (2016) Variation in the oral processing of everyday meals is associated with fullness and meal size; a potential nudge to reduce energy intake? Nutrients. 10.3390/nu8050315 PMC4882727 27213451

[28] Bolhuis DP, Forde CG (2020) Application of food texture to moderate oral processing behaviors and energy intake. Trends Food Sci Tech 106:445–456. 10.1016/j.tifs.2020.10.021

[29] Lasschuijt MP, Heuven LAJ, van Bruinessen M, Liu Z, Rubert J, Stieger M et al (2025) The effect of eating rate of ultra-processed foods on dietary intake, eating behaviour, body composition and metabolic responses—rationale, design and outcomes of the restructure randomised controlled trial. Nutr Bull 50(4):640–655. 10.1111/nbu.70027 PMC12621179 40926558

[30] Forde CG, Heuven LAJ, van Bruinessen M, Liu Z, Stieger M, de Graaf K et al (2025) Eating rate has sustained effects on energy intake from ultra-processed diets: a two-week ad libitum dietary randomized controlled cross-over trial. Am J Clin Nutr. 10.1016/j.ajcnut.2025.11.012 PMC13084570 41314613

[31] Cohen J (2013) Statistical power analysis for the behavioral sciences. Routledge

[32] Prater MC, Guadagni AJ, Cooper JA (2024) Postprandial appetite responses to a pecan enriched meal: a randomized crossover trial. Appetite 201:107598. 10.1016/j.appet.2024.107598 38971424

[33] Çelik MN, Ulug E (2025) Impact of ultra-processed foods on short-term appetite regulation: does body mass index make a difference? Int J Obes. 10.1038/s41366-025-01961-9 41429977

[34] Li J, Zhang N, Hu L, Li Z, Li R, Li C et al (2011) Improvement in chewing activity reduces energy intake in one meal and modulates plasma gut hormone concentrations in obese and lean young Chinese men123. Am J Clin Nutr 94(3):709–716. 10.3945/ajcn.111.015164 21775556

[35] van Strien T, Frijters JER, Bergers GPA, Defares PB (1986) The Dutch Eating Behavior Questionnaire (DEBQ) for assessment of restrained, emotional, and external eating behavior. Int J Eat Disord 5(2):295–315. 10.1002/1098-108X(198602)5:2<295::AID-EAT2260050209>3.0.CO;2-T

[36] Lucassen DA, Brouwer-Brolsma EM, Boshuizen HC, Mars M, de Vogel-Van den Bosch J, Feskens EJM (2023) Validation of the smartphone-based dietary assessment tool “Traqq” for assessing actual dietary intake by repeated 2-h recalls in adults: comparison with 24-h recalls and urinary biomarkers. Am J Clin Nutr 117(6):1278–1287. 10.1016/j.ajcnut.2023.04.008 37054887

[37] Lucassen DA, Brouwer-Brolsma EM, van de Wiel AM, Siebelink E, Feskens EJM (2021) Iterative Development of an Innovative Smartphone-Based Dietary Assessment Tool: Traqq (vol 169): 1940-087X 10.3791/62032 33818566

[38] Heuven LAJ, van Bruinessen M, Tang CS, Stieger M, Lasschuijt MP, Forde CG (2024) Consistent effect of eating rate on food and energy intake across twenty-four ad libitum meals. Br J Nutr 132(4):535–546. 10.1017/S0007114524001478 PMC11499084 39279635

[39] Lasschuijt M, Camps G, Mars M, Siebelink E, de Graaf K, Bolhuis D (2023) Speed limits: the effects of industrial food processing and food texture on daily energy intake and eating behaviour in healthy adults. Eur J Nutr 62(7):2949–2962. 10.1007/s00394-023-03202-z PMC10469122 37452167

[40] van Bruinessen M, Heuven LAJ, Stieger M, Lasschuijt MP, Forde CG (2026) The sustained effect of texture-based eating rate on food intake in an 11-d randomised controlled trial. Br J Nutr. 10.1017/S0007114525106193 PMC13315526 41492689

[41] Lasschuijt MP, Heuven LAJ, Gonzalez-Estanol K, Siebelink E, Chen Y, Forde CG (2025) The effect of energy density and eating rate on ad libitum energy intake in healthy adults—a randomized controlled study. J Nutr 155(8):2602–2610. 10.1016/j.tjnut.2025.06.006 40516651

[42] Flint A, Raben A, Blundell JE, Astrup A (2000) Reproducibility, power and validity of visual analogue scales in assessment of appetite sensations in single test meal studies. Int J Obes 24(1):38–48. 10.1038/sj.ijo.0801083 10702749

[43] Allison DB, Paultre F, Maggio C, Mezzitis N, Pi-Sunyer FX (1995) The use of areas under curves in diabetes research. Diabetes Care 18(2):245–250. 10.2337/diacare.18.2.245 7729306

[44] Blundell J, De Graaf C, Hulshof T, Jebb S, Livingstone B, Lluch A et al (2010) Appetite control: methodological aspects of the evaluation of foods. Obes Rev 11(3):251–270. 10.1111/j.1467-789X.2010.00714.x PMC3609405 20122136

[45] Higgs S (2008) Cognitive influences on food intake: the effects of manipulating memory for recent eating. Physiol Behav 94(5):734–739. 10.1016/j.physbeh.2008.04.012 18486159

[46] Higgs S (2002) Memory for recent eating and its influence on subsequent food intake. Appetite 39(2):159–166. 10.1006/appe.2002.0500 12354684

[47] Brunstrom JM, Brown S, Hinton EC, Rogers PJ, Fay SH (2011) Expected satiety’ changes hunger and fullness in the inter-meal interval. Appetite 56(2):310–315. 10.1016/j.appet.2011.01.002 21219951

[48] Willian AR, Pater JL (1986) Using baseline measurements in the two-period crossover clinical trial. Control Clin Trials 7(4):282–289. 10.1016/0197-2456(86)90036-X 3802851

[49] McCambridge J, Witton J, Elbourne DR (2014) Systematic review of the Hawthorne effect: new concepts are needed to study research participation effects. J Clin Epidemiol 67(3):267–277. 10.1016/j.jclinepi.2013.08.015 PMC3969247 24275499

[50] Hall KD, Ayuketah A, Brychta R, Cai H, Cassimatis T, Chen KY et al (2019) Ultra-processed diets cause excess calorie intake and weight gain: an inpatient randomized controlled trial of ad libitum food intake. Cell Metab 30(1):67-77.e63. 10.1016/j.cmet.2019.05.008 PMC7946062 31105044

[51] King JA, Thackray AE, Gibbons C, Martins C, Broom DR, Stensel DJ et al (2025) The mixed-meal tolerance test as an appetite assay: methodological and practical considerations. Int J Obes 49(11):2168–2183. 10.1038/s41366-025-01866-7 PMC12583139 40739367

[52] Galdino-Silva MB, Almeida KMM, Oliveira ADSd, Santos JVLd, Macena MdL, Silva DR et al (2024) A meal with ultra-processed foods leads to a faster rate of intake and to a lesser decrease in the capacity to eat when compared to a similar, matched meal without ultra-processed foods. Nutrients 16(24):4398. 10.3390/nu16244398 PMC11676177 39771019

[53] Pinson A, Glachet C, Jacquinet C, Sevrin E, Moralia M-A, Parent A-S (2025) Endocrine disrupting chemicals alter neuronal circuits controlling reproduction and appetite. Ann Endocrinol (Paris) 86(3):101781. 10.1016/j.ando.2025.101781 40339691

[54] Bergman A, Heindel JJ, Kasten T, Kidd KA, Jobling S, Neira M et al (2013) The impact of endocrine disruption: a consensus statement on the state of the science. Environ Health Perspect 121(4):A104-106. 10.1289/ehp.1205448 PMC3620733 23548368

[55] Cummings DE (2006) Ghrelin and the short- and long-term regulation of appetite and body weight. Physiol Behav 89(1):71–84. 10.1016/j.physbeh.2006.05.022 16859720

[56] Aukan MI, Coutinho S, Pedersen SA, Simpson MR, Martins C (2023) Differences in gastrointestinal hormones and appetite ratings between individuals with and without obesity—a systematic review and meta-analysis. Obes Rev 24(2):e13531. 10.1111/obr.13531 PMC10078575 36416279

[57] Tschop M, Weyer C, Tataranni PA, Devanarayan V, Ravussin E, Heiman ML (2001) Circulating ghrelin levels are decreased in human obesity. Diabetes 50(4):707–709. 10.2337/diabetes.50.4.707 11289032

[58] Greenway FL (2015) Physiological adaptations to weight loss and factors favouring weight regain. Int J Obes 39(8):1188–1196. 10.1038/ijo.2015.59 PMC4766925 25896063

[59] Jin Z, Li J, Thackray AE, Shen T, Deighton K, King JA et al (2025) Fasting appetite-related gut hormone responses after weight loss induced by calorie restriction, exercise, or both in people with overweight or obesity: a meta-analysis. Int J Obes (Lond) 49(5):776–792. 10.1038/s41366-025-01726-4 PMC12095072 39929932

[60] Sumithran P, Prendergast LA, Delbridge E, Purcell K, Shulkes A, Kriketos A et al (2011) Long-term persistence of hormonal adaptations to weight loss. N Engl J Med 365(17):1597–1604. 10.1056/NEJMoa1105816 22029981

[61] Teo PS, Lim AJ, Goh AT, Janani R, McCrickerd K, Forde CG (2026) Impact of food texture and degree of food processing on post-meal satiety and later snack intake. Appetite 225:108604. 10.1016/j.appet.2026.108604 42176807

[62] Scrinis G, Popkin BM, Corvalan C, Duran AC, Nestle M, Lawrence M et al (2025) Policies to halt and reverse the rise in ultra-processed food production, marketing, and consumption. Lancet 406(10520):2685–2702. 10.1016/S0140-6736(25)01566-1 41270767

[63] Chambers L, McCrickerd K, Yeomans MR (2015) Optimising foods for satiety. Trends Food Sci Tech 41(2):149–160. 10.1016/j.tifs.2014.10.007

