# Supplementary material for: Satiety responsiveness following dietary exposure to ultra-processed foods differing in texture-derived eating rate: a secondary analysis of a randomized controlled crossover diet intervention
Source: Eur J Nutr. 2026 Sep 24;65(7):261. doi: 10.1007/s00394-026-04111-7 (PMC13612719; doi:10.1007/s00394-026-04111-7)
Supplement: Supplementary file 1 [file 394_2026_4111_MOESM1_ESM.docx]

**Satiety responsiveness following dietary exposure to ultra-processed foods differing in texture-derived eating rate: a secondary analysis of a randomized controlled crossover diet intervention**

Marieke van Bruinessen^1^, Zhen Liu^1^, Lise A.J. Heuven^1^, Markus Stieger^1^, Marlou P. Lasschuijt^1^, Ciarán G. Forde^1^

^1^Sensory Science and Eating Behavior Group, Division of Human Nutrition and Health, Wageningen University & Research, Wageningen, the Netherlands

**Supplementary Table 1**

**Table 1** Detection range, and intra- and inter-assay coefficients of variation (CVs) for satiety hormone analysis.

|  | **Detection range** | **Intra-assay CV (%)** | | **Inter-assay CV (%)** |
| --- | --- | --- | --- | --- |
| Ghrelin (total) | 1.7 – 2,710 pg/mL | 6.9 | 38 | |
| Glucagon-like peptide-1 | 0.59 – 576 pM | 4.9 | 17 | |
| Peptide YY (total) | 2.7 – 2,260 pg/mL | 6.7 | 35 | |
| Pancreatic polypeptide | 0.19 – 1,830 pg/mL | 2.7 | 9 | |

**Supplementary Table 2**

**Table 2** Daily estimated dietary intake (based on a 3-day food diary) at baseline and actual weighted dietary intake for the two UPF diets. Data are presented as mean (95% CI).

|  | **Baseline**  **(Estimated intake)^a^** | **Slow UPF**  **(Measured intake)^b^** | **Fast UPF**  **(Measured intake)^b^** |
| --- | --- | --- | --- |
| Daily energy intake (kcal/day) | 2044 (1832-2257) | 2301 (2042-2562) | 2671 (2411-2930) |
| Daily food intake (g/day) | 2655 (2289-3022) | 1481 (1324-1639) | 1746 (1588-1904) |
| Carbohydrate (g), EN% | 207 (182-234), 41% | 310 (279-340), 54% | 320 (289-350), 48% |
| Monosaccharides and disaccharides (g) | 75 (64-86) | 89 (77-101) | 130 (118-142) |
| Fat (g), EN% | 90 (79-100), 39% | 60 (52-68), 24% | 99 (91-108), 33% |
| Saturated fat (g) | 29 (26-33) | 15 (13-18) | 39 (37-42) |
| Protein (g), EN% | 86 (75-96), 18% | 110 (99-122), 19% | 103 (92-115), 15% |
| Fiber (g) EN% | 23 (19-27), 2% | 36 (33-39), 3% | 39 (36-42), 3% |
| Sodium intake (mg/100 kcal) | 1.1 (0.9-1.2) | 3.8 (3.4-4.2) | 4.2 (3.8-4.7) |
| Daily alcohol intake (g) | 3.3 (0.9-5.8) | NA | NA |

Abbreviations: CI, confidence interval; EN, Energy; UPF, ultra-processed food.

^a^Estimated habitual dietary intake measured using a 3-day food diary.

^b^Measured intake during the UPF diets with all food weighed to the nearest gram.
